# Fluorescent Nanodiamonds for Tracking Single Polymer Particles in Cells and Tissues

**DOI:** 10.1021/acs.analchem.3c01452

**Published:** 2023-08-23

**Authors:** Runrun Li, Thea A. Vedelaar, Alina Sigaeva, Yue Zhang, Kaiqi Wu, Hui Wang, Xixi Wu, Peter Olinga, Małgorzata K. Wlodarzyk-Biegun, Romana Schirhagl

**Affiliations:** † Department of Biomedical Engineering, Groningen University, University Medical Center Groningen, Antonius Deusinglaan 1, 9713AV Groningen, The Netherlands; ‡ Zernike Institute for Advanced Materials, Groningen University, Nijenborgh 4, 9747 AG Groningen, The Netherlands; § Department of Pharmaceutical Technology and Biopharmacy, Groningen University, University Medical Center Groningen, Antonius Deusinglaan 1, 9713AV Groningen, The Netherlands; ∥ Biotechnology Centre, The Silesian University of Technology, Krzywoustego 8, 44-100 Gliwice, Poland

## Abstract

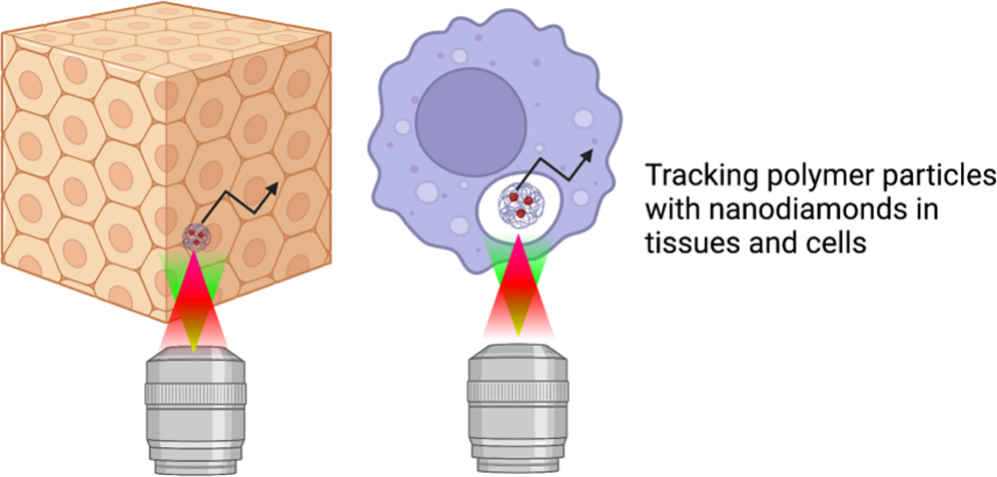

Polymer nanoparticles are widely used in drug delivery and are also a potential concern due to the increased burden of nano- or microplastics in the environment. In order to use polymer nanoparticles safely and understand their mechanism of action, it is useful to know where within cells and tissues they end up. To this end, we labeled polymer nanoparticles with nanodiamond particles. More specifically, we have embedded nanodiamond particles in the polymer particles and characterized the composites. Compared to conventional fluorescent dyes, these labels have the advantage that nanodiamonds do not bleach or blink, thus allowing long-term imaging and tracking of polymer particles. We have demonstrated this principle both in cells and entire liver tissues.

## Introduction

Polymer nanoparticles are widely used for drug delivery.^1−3^ It is important to understand their interactions with biological samples to assess nanoparticle toxicity.^4^ Further, it is essential to know how polymer particles, produced by degrading plastic waste in the environment, interact with biological matter.^5^ For all of these applications, it is vital to study where within cells or tissues polymer nanoparticles exactly end up. There are several imaging approaches to visualize polymer particles.^6^ Electron microscopy is an attractive tool to achieve this goal.^7^ However, electron microscopy is usually not compatible by analyzing living samples. Additionally, most polymers are relatively similar in elemental composition to biological matter, and thus, this is especially challenging for small particles. Optical microscopy and spectroscopy offer an alternative, but most polymer particles cannot be detected directly with these approaches either.^8,9^ To circumvent this problem, it is possible to include fluorescent labels inside the polymer. However, conventional fluorescent labels suffer from bleaching and can thus not be followed in the long term.

Fluorescent nanodiamonds (FNDs) offer an attractive alternative to conventional fluorescent labels. FNDs are excellently biocompatible in different kinds of cells and even in entire organisms.^10−13^ Further, FNDs are inert but can be chemically functionalized via their rich surface chemistry. Most importantly, FNDs can host color centers that are protected within the diamond crystal lattice and emit infinitely stable fluorescence. Making use of these properties, FNDs have been utilized for long-term fluorescent labeling as well as tracking.^14−17^ By attaching antibodies, glycans, or targeting peptides, it is possible to target nanodiamonds to specific regions within the cell or tissue to visualize these structures.^18−20^ It is also attractive that FNDs are well visible in different imaging techniques, which are interesting for correlative microscopy.^21^ Finally, nanodiamonds can be used for quantum sensing to determine the particle orientation,^22^ temperature,^23,24^ or the free radical load in their surroundings.^25−28^ Here, we use these FNDs for the first time to track where polymer particles reside within cells or tissues.

**Figure 1:**
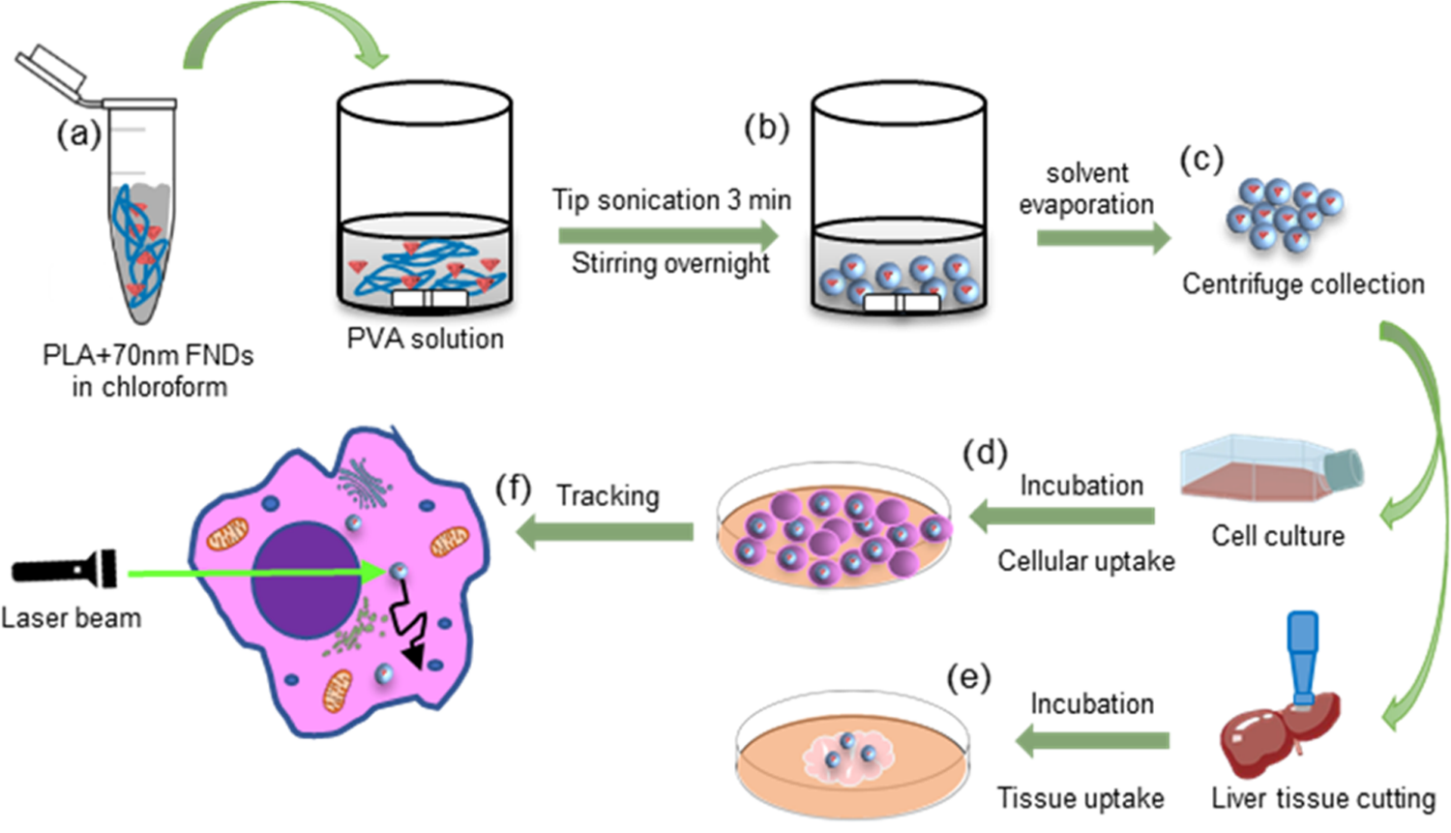
Preparation of PLA-FND particles, *in vitro* evaluation, and cellular tracking. (a) PLA and FNDs in chloroform are added to PVA in aqueous solution. (b) Tip sonication facilitates the formation of an emulsion, and the presence of PVA stabilizes the emulsion and leads to a uniform particle size distribution. (c) Particles were collected by centrifugation after magnetic stirring overnight. (d) Particle internalization and location within the cell and quantitative analysis. (e) Particle internalization in mouse liver slice. (f) Diamonds within polymer particles were tracked using a custom-built confocal microscope.

The study scheme, including particle preparation and *in vitro* measurements, is shown in Figure 1.

## Materials and Methods

Throughout this study, we use FNDs of an average hydrodynamic diameter of 70 nm (commercially available from Adamas Nanotechnologies). These FNDs are produced by high-pressure high-temperature synthesis by the manufacturer as described before.^29^ After that, they are irradiated with high-energy electrons at 3 MeV and a fluence of 5 × 10^19^ e/cm^2^, followed by a high-temperature annealing step. Finally, FNDs are cleaned by the manufacturer in oxidizing the acid to obtain oxygen-terminated FNDs. The diamond particles themselves are widely used and well characterized in the field.^30,29^

Polylactic acid (PLA, Mw 60,000), chloroform (≥99.5%), poly(vinyl alcohol) (PVA,87–90% hydrolyzed, average mol wt 30,000–70,000), 4,6- diamidino-2-phenylindole (DAPI), phalloidin–fluorescein isothiocyanate labeled (phalloidin-FITC), and lysosome-associated membrane protein 1(LAMP-1, AB2971) were all purchased from Sigma-Aldrich, The Netherlands. 4% formaldehyde solution was obtained from Klinipath, Poland, and early endosomal antigen 1 (EEA1, # MA5-14794) from Thermofisher, The Netherlands. Donkey-α-rabbit IgG FITC was purchased from Jackson, U.K. (711-095-152). Dulbecco’s Modified Eagle Medium with high glucose (DMEM-HG, 31966047), fetal bovine serum (FBS, 10270106), and penicillin–streptomycin (10,000 U/mL, 15140122) were obtained from Gibco, The Netherlands. The adherent mouse macrophage cell line, J774, was obtained from ATCC/LCG and cultured in DMEM-HG supplemented with 10% (v/v) FBS, 100 units/mL penicillin, and 100 mg/mL streptomycin in a humidified 5% CO_2_/95% air atmosphere at 37 °C.

### Synthesis and Characterization of nanoparticles

PLA nanoparticles and PLA NPs loaded with FNDs (PLA-FNDs) were prepared by a classic solvent emulsion evaporation method.^31^ Appropriate amounts of PLA were dissolved in chloroform to 10 mg/mL, and PVA was dissolved in Milli-Q water at 60 °C to 1% w/v, which is used here as the dispersant to control particle size and reduce aggregation. Then, the PLA solution was added to an aqueous solution of PVA (1:6 v/v PLA solution and PVA solution). For PLA nanoparticles loaded with FNDs, 70 nm FNDs (2%, w(FNDs)/w(PLA)) were mixed with the PLA solution in chloroform first and then added into the aqueous solution of PVA. The mixture was tip-sonicated (Vibra-Cells, VCX130, 2 mm stepped micro tip) for 3 min in a pulsed manner and 40% amplitude over an ice bath. The emulsion was magnetically stirred overnight, while chloroform was evaporated at room temperature. Subsequently, nanoparticles were collected by centrifugation at 10,000 rpm for 15 min in a pellet. We removed the solvent and resuspended the particles in a Milli-Q water. To wash the particles and remove residual PVA, we centrifuged again and removed the solvent. Washing was repeated 3 times in total. Nanoparticles were resuspended in water and stored at 4 °C.

The morphology of the nanoparticles was observed with a scanning electron microscope (SEM, FEI Nova NanoSEM 650). We dropped 10 μL of a 2 mg/mL nanoparticle suspension onto small silicon wafers and left the samples to dry. Images were taken under high vacuum at an acceleration of 10 kV. Particle size distributions and zeta potentials were determined by a Zetasizer Nano ZS ZEN3600 system (Malvern Instruments, U.K.) at 25 °C (see the Supporting Information). The biocompatibility of the particles was confirmed using an XTT assay (Supporting Information).

### Cellular Uptake of FNDs and PLA-FND Nanoparticles

J774 cells were seeded in a 35 mm Petri dish with four compartments after reaching 70–90% confluency at a density of 5000 cells/compartment and left for 24 h. Cells were incubated with 4 μg/mL FNDs or 200 μg/mL PLA-FNDs (the same amount of FNDs as in PLA-FNDs theoretically) for 2 h. Then, we replaced the medium with fresh medium without particles and incubated for 0, 6, and 24 h. The cells incubated with PLA nanoparticles and cells without particles served as controls. Cells were fixed with 4% paraformaldehyde for 15 min. Fixed cells were permeabilized with 0.5% Triton X-100 in PBS for 3 min. Then, the cytoskeleton was stained with 2 μg/mL of phalloidin-FITC for 30 min in the dark. Nuclei were stained with 2 μg/mL DAPI for 10 min. Cells were stored at 1% paraformaldehyde for imaging. Nanoparticles were visualized by using a Zeiss LSM780 confocal microscope. Filter settings for DAPI, phalloidin-FITC, and FNDs were 408/461, 488/525, and 561/650 nm, respectively.

### Tissue Slicing and Uptake of FNDs and PLA-FND Nanoparticles

Animal experiments and the use of tissue were approved by the Animal Ethical Committee of the University of Groningen (CCD AVD10500202216104) and were performed in accordance with EU Directive 2010/63/EU for animal experiments. The protocol of precision-cut liver slices was already described in previous work.^32^ Briefly, excised mouse liver and cylindrical cores were stored at the University of Wisconsin (UW) organ preservation solution (DuPont Critical Care, Illinois). The cores were prepared by using a 5 mm biopsy punch. Approximately 250 μm thick slices were prepared using a Krumdieck Live Tissue Microtome-MD6000 (Alabama Research and Development, Munford, AL). Ice-cold Krebs–Henseleit buffer 1× supplemented with 25 mM d-glucose (Merck, Darmstadt, Germany), 25 mM NaHCO_3_ (Merck), and 10 mM 4-(2-hydroxyethyl) piperazine-1-ethanesulfonic acid (MP Biomedicals GmbH, Germany) saturated with 95% O_2_ and 5% CO_2_ were used to collect slices. Then, liver slices were cultured in William’s E medium with GlutaMAX (Life Technologies, Carlsbad) supplemented with 2.75 μg/mL d-glucose monohydrate (Merck, Darmstadt, Germany) and 50 μg/mL gentamicin (Invitrogen, Paisley, U.K.) at 37 °C and 5% CO_2_ and 80% O_2_. 50 μg of FNDs and 2.5 mg of PLA-FNDs (the same amount of FNDs in PLA-FNDs as for bare FNDs) were added separately to 500 μL William’s E medium in each well of a 12-well plate. Liver slices were incubated with the medium containing a suspension of particles up to 48 h, and this medium was refreshed every 24 h. Liver slices were fixed for 15 min with 2% formaldehyde and imaged using a Zeiss LSM780 confocal microscope connected with a two-photon laser at an 850 nm excitation wavelength.

### Cellular Distribution of FNDs and PLA-FND Nanoparticles

To verify the intracellular distribution of FNDs and PLA-FND nanoparticles, endosome and lysosome staining were performed. The same protocol as that described above was used for cellular uptake. After incubation with FNDs and PLA-FNDs for 0, 6, and 24 h, the cells were fixed and washed with cold PBS three times. Then, cells were permeabilized with 0.5% Triton X-100 for 15 min and washed with PBS three times for 5 min each. 1% BSA (bovine serum albumin) in PBS (PBSA) was added to cells as a blocking agent to bind nonspecific sites that interfere with antigen binding. Afterward, cells were stained with EEA1 in PBSA (early endosome marker, dilution 1:200) and LAMP-1 in PBSA (late endosomes and lysosomes marker, dilution 1:500) for 1 h at room temperature in the dark. Cells were washed with PBS three times for 5 min each and incubated with a secondary antibody (Donkey-α-rabbit IgG FITC in PBSA) for both primary antibodies (dilution 1:200). The cells were incubated for 1 h at room temperature in the dark. Then, cells were washed with PBS three times for 5 min each again and stored at 1% paraformaldehyde for imaging (Zeiss LSM700 confocal microscope). Filter settings of FITC and FNDs were 488 nm/525 nm and 561/650 nm, respectively.

### Imaging Acquisition and Processing

3D z-stack images were obtained with a 63 × 1.3 oil objective, and all fluorescence signals were collected with 200 × 200 × 200 nm^3^ per pixel to record particle internalization and their spatial distribution. All images of cells were deconvoluted by two plugins of FIJI software: the “PSF generator” and “DeconvolutionLab2”. The “PSF generator” plugin is used to generate a realistic point spread function (PSF) and correct blurred representations of the actual object due to diffraction. The “DeconvolutionLab2” plugin is a mathematical inverse method that represents the original signal of imaging by making use of the PSF. The ″3D object counter” plugin is used to calculate the number of intracellular particles in z-stack images. The deconvolved images of the EEA1 and LAMP-1 channels were transformed into 3D Euclidean distance maps of the proteins. If the minimum distance of particles from the 3D Euclidean distance map was 0, then the particles were considered to be colocalized with the endosomes or lysosomes. All image analyses were performed using FIJI software.

#### Statistics

For all experimental data, we performed statistical evaluation by GraphPad Prism 8.0. To determine statistical significance, we used the Kruskal–Wallis test of one-way ANOVA to compare three or more groups. Data were presented as the mean ± standard deviation. The statistical results are shown as *p ≤ 0.05, **p ≤ 0.01, ***p ≤ 0.001, ****p ≤ 0.0001, and ns indicates nonsignificance. Confocal images were observed and analyzed by ZEN 3.3 and FIJI software.

## Results and Discussion

### Cellular and Tissue Uptake

**Figure 2:**
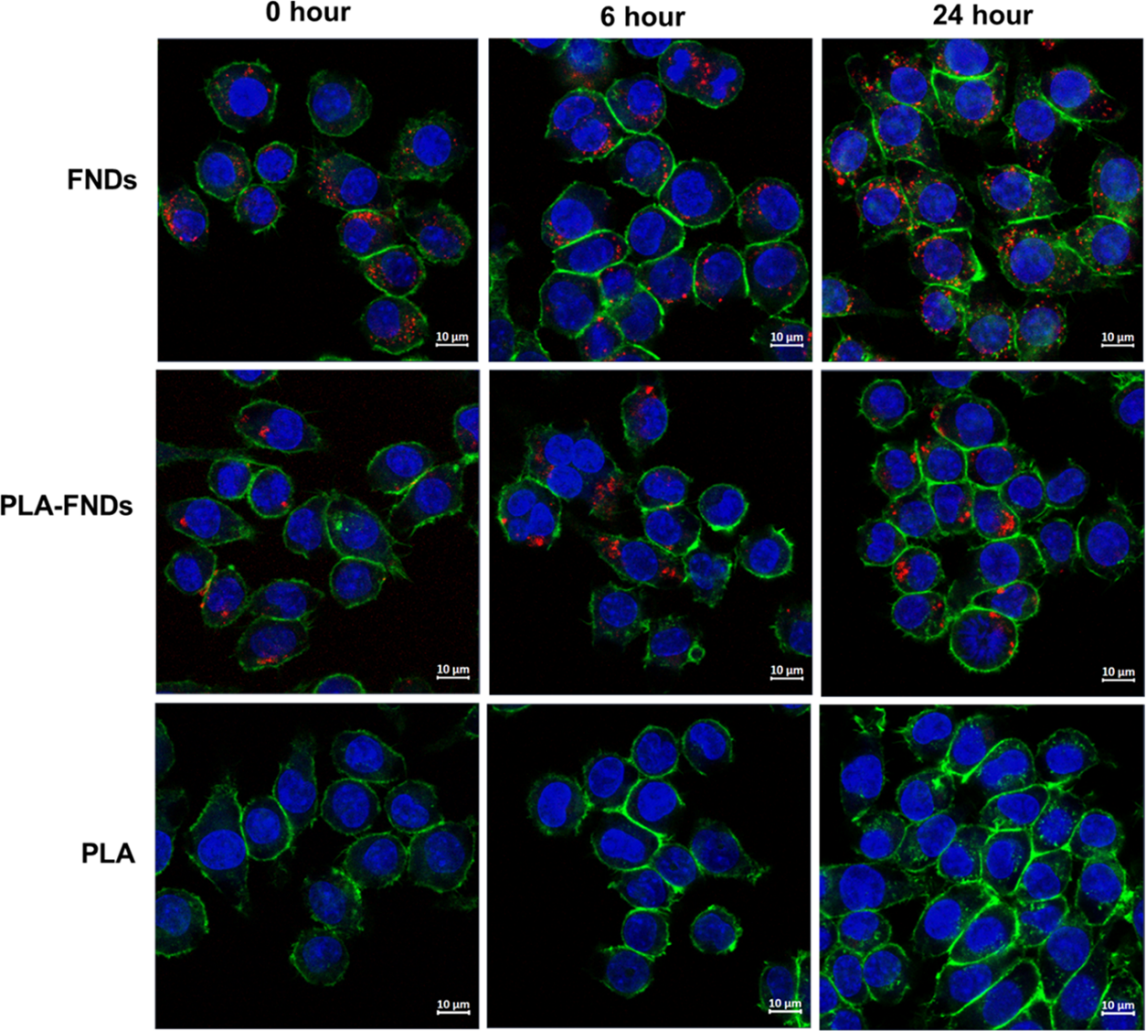
FNDs, PLA-FNDs, and PLA nanoparticles were taken up by macrophages after 2 h of incubation with particles plus 0, 6, and 24 h of incubation in the medium without particles. Red represent the FNDs, green represents the cytoskeleton, and blue represents the nucleus. Merged images are shown. The red fluorescence of FNDs and PLA-FNDs was seen after nanoparticles were taken up. In the PLA samples, we did not observe any red fluorescence inside the cells, indicating that PLA itself has no autofluorescence across the visible wavelength we used.

To study the interaction between PLA and the cells, we incubated macrophages with FND-PLA and FND for comparison. Figure 2 shows the results of these experiments. Already from these images, it is clear that macrophages ingest both FNDs and PLA-FNDs. When we compare these images with the control with PLA only, we do not see any red particles. This indicates that the particles that we see are indeed FNDs. This can be further confirmed by the fact that FNDs do not bleach and show perfectly stable fluorescence.

**Figure 3:**
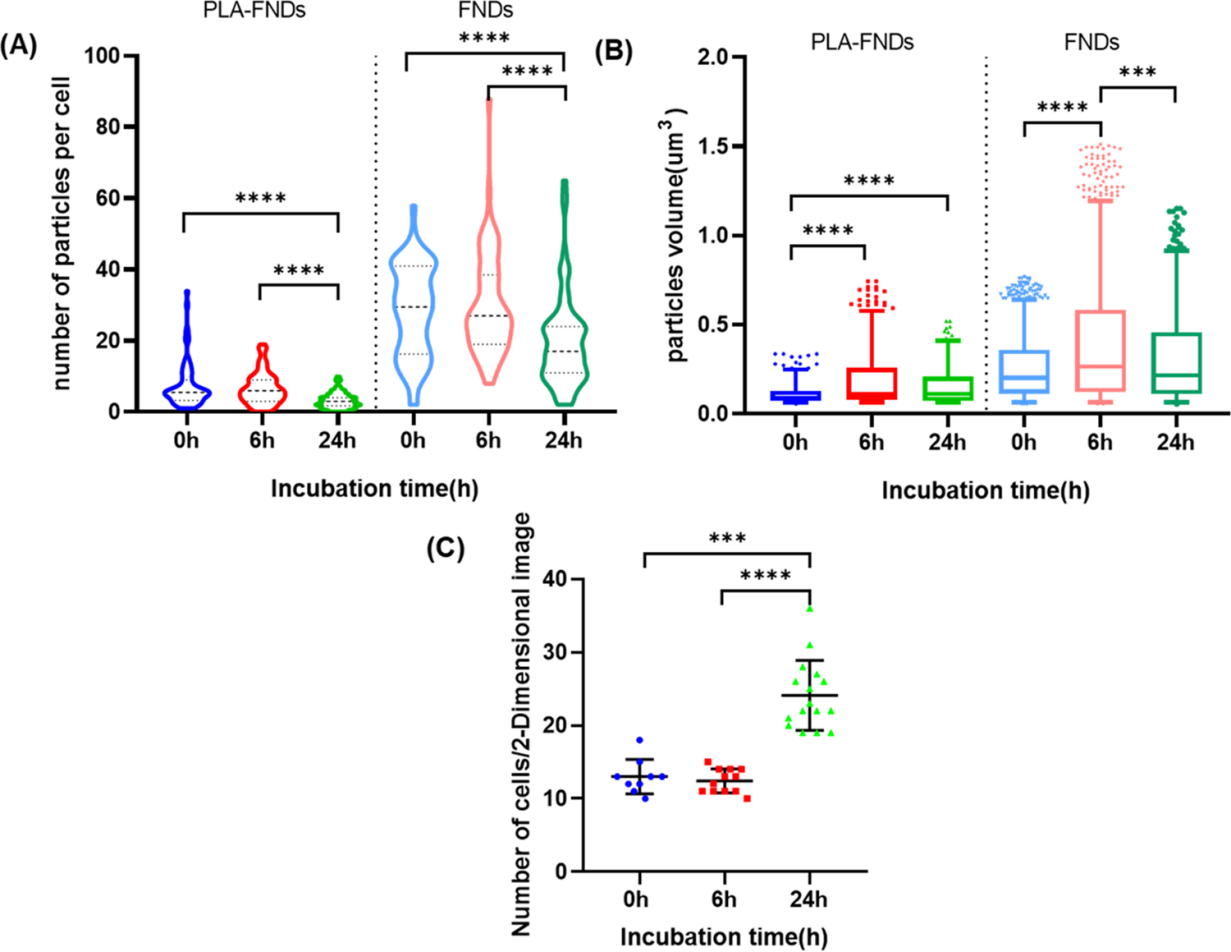
Number of particles in each cell decreases over time; however, noticeable aggregation occurred. All data were analyzed from at least 50 cells. (A) The spatial distribution of particles per cell is shown in violin plots. Wider sections mean a higher probability that the number of particles per cell is distributed there. Black dotted lines from top to bottom mean third quartile, median, and first quartile, respectively. (B) The particle volume is shown in box plots. The box covers the 5–95 percentile of the data, and the horizontal lines that split the box are the median. (C) Cell numbers at varying incubation times are shown in scatter plots. Black lines are mean ± standard deviation.

To quantify the particle numbers that we see per cell, we performed particle counting. The results are shown in Figure 3A. From this experiment, we can draw several conclusions. The number of FNDs or FND-PLA molecules per cell decreases with time. Further, we can see that FNDs are ingested to a larger extent than FND-PLA. Many studies have demonstrated that particle size is one of the key factors for cellular uptake. Some studies indicated that higher uptake was observed for smaller nanoparticles and medium uptake for medium-sized particles.^33^ However, some studies also indicated that smaller or larger particles could decrease the uptake rate; there is an optimum size, around 50 or 100 nm, where the uptake rate is the highest. In Figure 3A, there was no significant difference in the number of particles per cell either PLA-FNDs or FNDs between 0 and 6 h, but there was a significant difference between the early time points and 24 h. The number of cells barely increased during incubation from 0 to 6 h, but there was a significant increase after incubation of 24 h, with approximately twice as many cells in the observed region as after 0 h and 6 h, as shown in Figure 3C. Studies have shown that particles are divided among daughter cells during the cell division, which is likely responsible for the number of particles per cell decreasing after 24 h incubation.^34,35^Figure 3B demonstrates the aggregation of the particles inside the cell by analyzing the particle volume. We found that FNDs aggregate (or otherwise end up in the same location) more inside the cell than PLA-FNDs, as the volume of FND aggregation is approximately twice that of PLA-FNDs. This is probably due to the transport and storage of particles by the cells in the same locations. Here, it has to be noted that the volumes that are shown are only from the bright part of the particle (the polymer itself is invisible) since the PLA part of PLA-FNDs is invisible. Aggregation of either PLA-FNDs or FNDs was most notable at 6 h, and the volume of PLA-FNDs did not change significantly after 24 h of incubation, but the volume of FNDs became significantly smaller. FNDs have been shown to undergo aggregation when they are exposed to the medium or end up together when they are ingested in endosomes and/or lysosomes, but aggregation does not affect the NV center fluorescence of each diamond.^36^ Polymer particles might also aggregate due to the surface energy or particle size. Here, only polymer nanoparticles loaded with nanodiamonds were considered because nanodiamonds have unique optical stability for long-term tracking.

**Figure 4:**
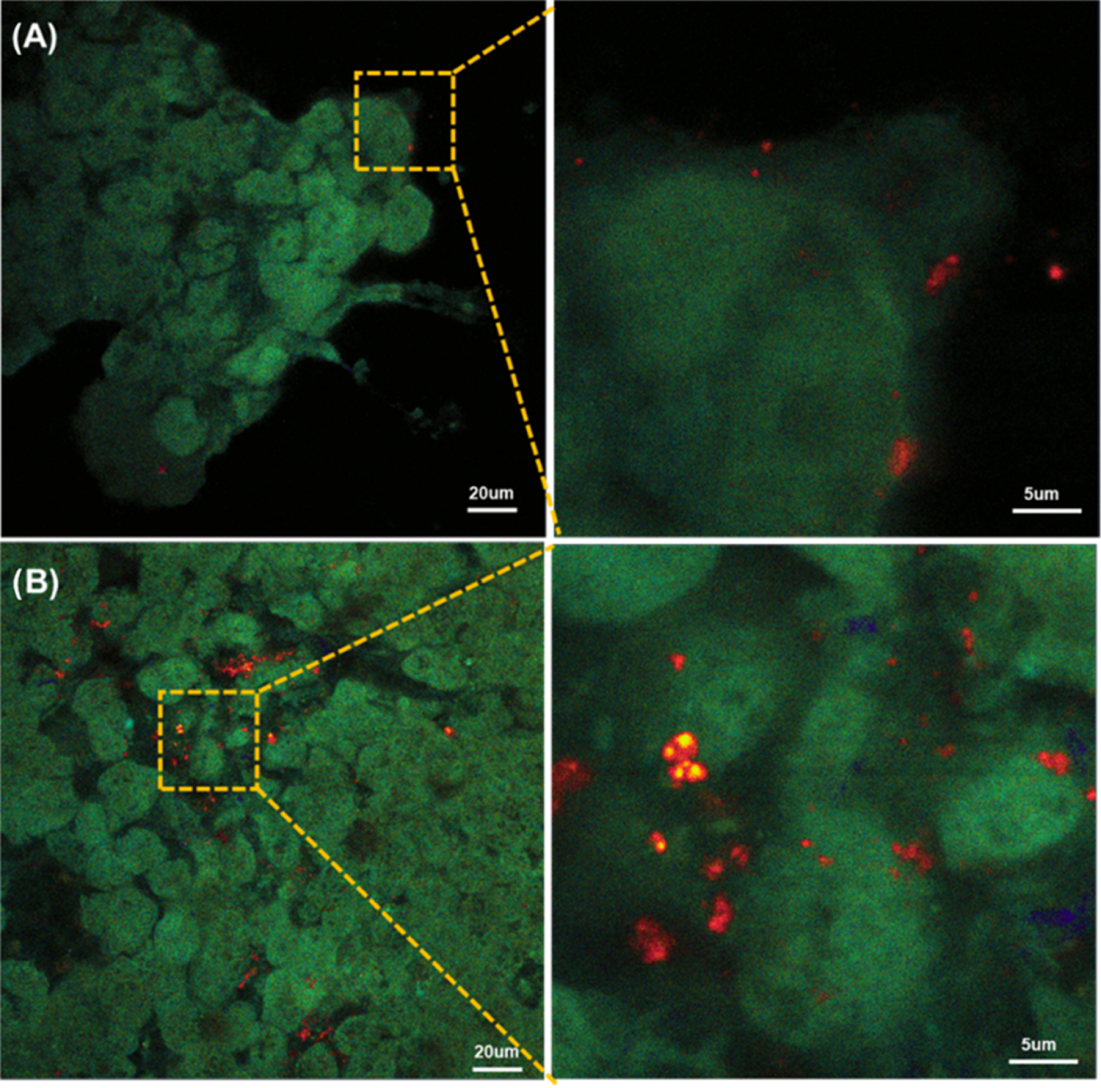
PLA-FNDs (A) and FNDs (B) were also observed in mouse liver tissue after 48 h of incubation (red: FNDs, green: tissue autofluorescence).

To demonstrate labeling in tissue samples, we used 250 μm thick precision-cut liver slices (PCLS). These are an attractive alternative to experiments on living animals, which are limited for ethical reasons. If slices are used, then one can test multiple conditions in slices from the same animal. This reduces the differences in biological variability and reduces the number of required animals. Images were taken by two-photon microscopy, which has a longer wavelength and a lower energy but deeper tissue penetration than single-photon measurements. Figure 4 shows that both FNDs and PLA-FNDs can be taken up by the PCLS. Also, in tissue, FNDs seem to be easier to detect and more numerous. Red fluorescence of FNDs from PLA-FNDs was observed both at the edges and inside the tissue. Green fluorescence is tissue autofluorescence because of endogenous tissue components, such as the aromatic amino acids, collagens, nicotinamide adenine dinucleotide, retinol, and riboflavin. The concentrations of those intrinsic fluorophores are extremely high in tissue depending on tissue type.^37,38^ The results reveal that 2% of FNDs is enough to track PLA nanoparticles in tissue. Apart from what we have shown here, FNDs may also be used as a tool to track the polymer degradation in real time by quantum sensing.^39^

### Intracellular Location of Nanoparticles

**Figure 5:**
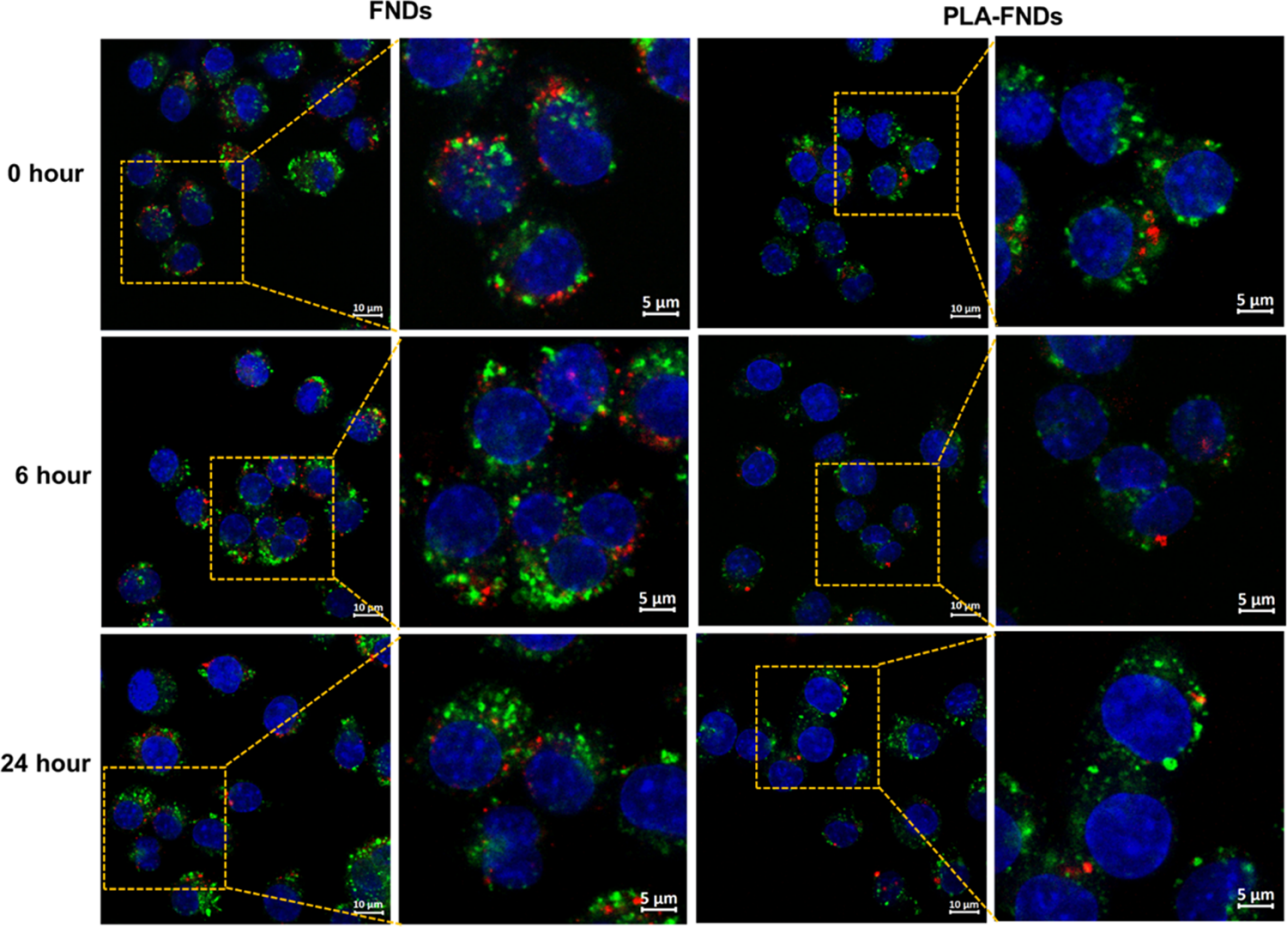
Colocalization of PLA and PLA-FNDs with early endosome compartments labeled by EEA1 antibody after 0, 6, and 24 h of incubation (red: FNDs, green: early endosomes, blue: nucleus). Merged images are shown. All data were analyzed from at least 50 cells.

**Figure 6:**
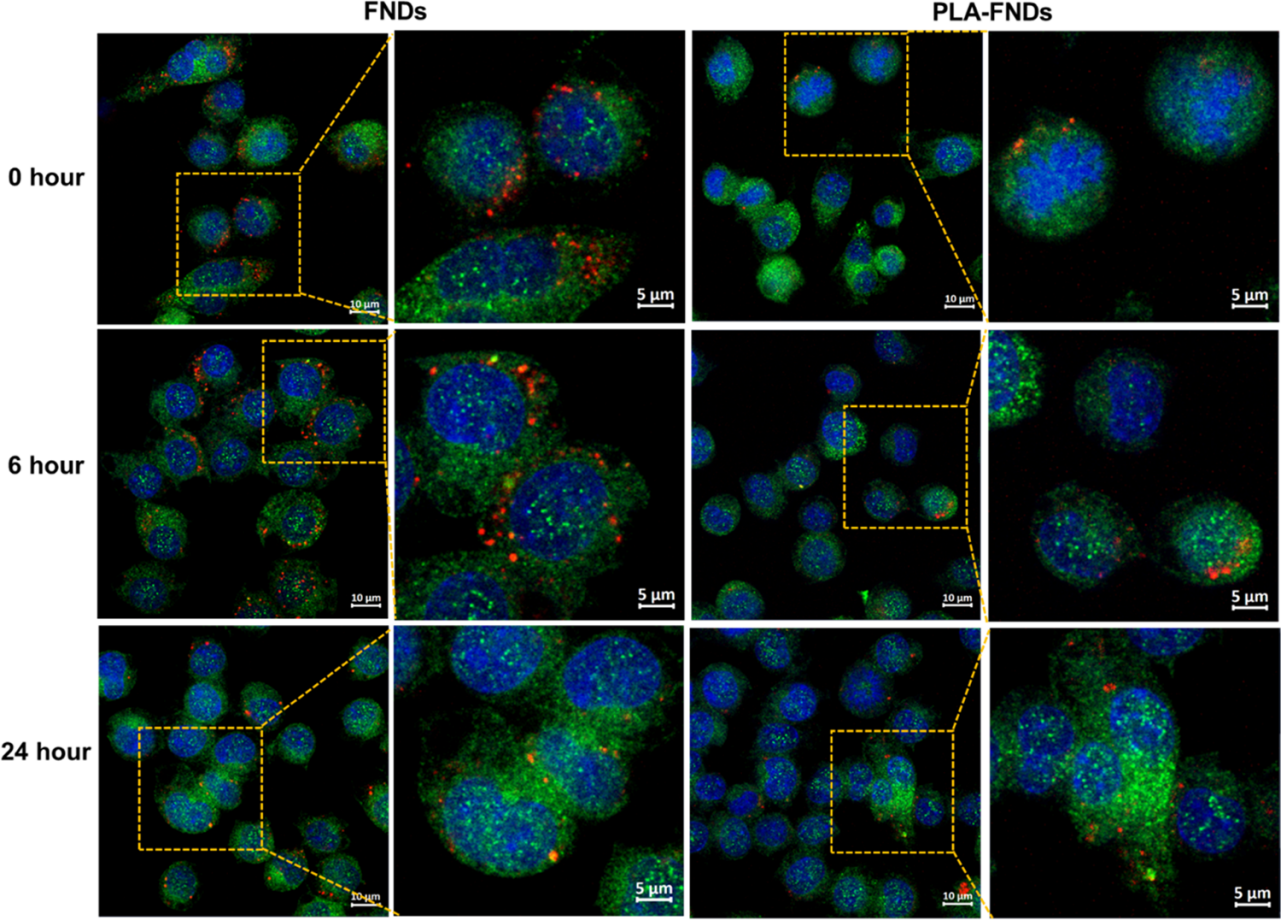
Colocalization of FNDs and PLA-FNDs with lysosome compartments labeled by LAMP-1 antibodies after 0, 6, and 24 h of incubation (red: FNDs, green: late endosomes and lysosomes, blue: nucleus). Merged images are shown. All data were obtained from at least 50 cells.

**Figure 7:**
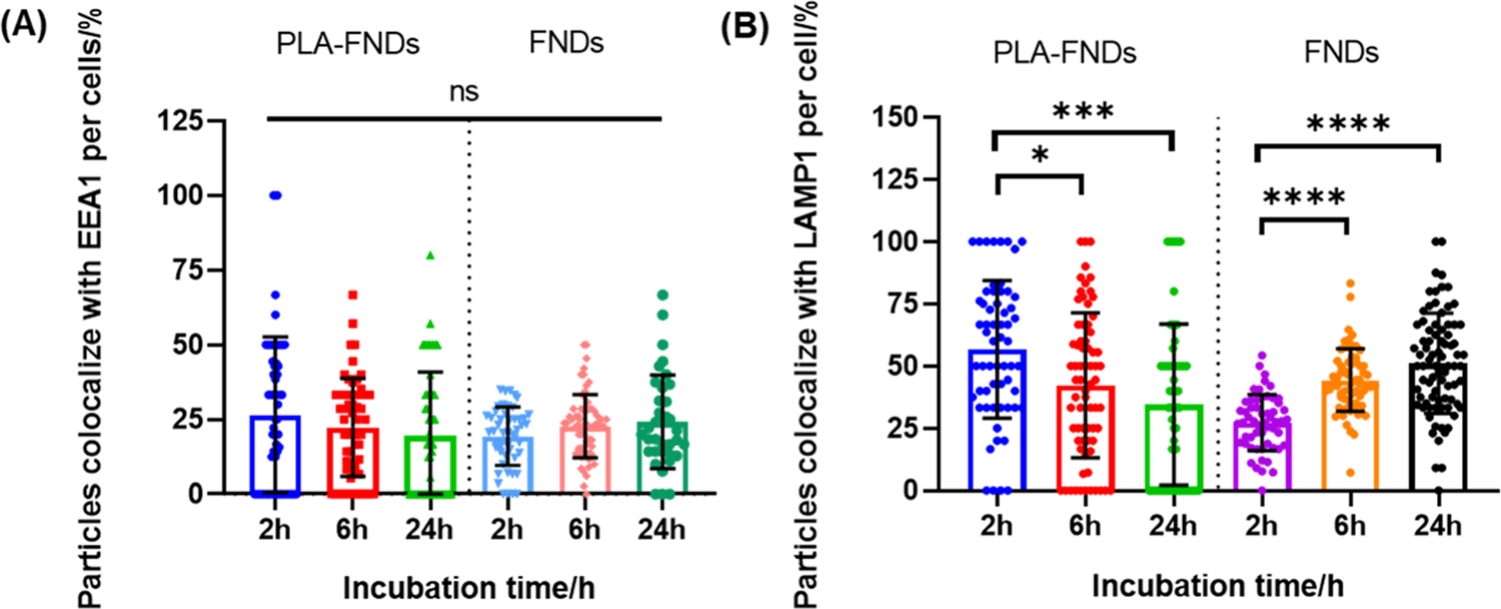
Quantitative colocalization analysis of ingested FNDs and PLA-FNDs with endosomes and lysosomes from confocal microscopy images. All data are shown as mean ± standard deviation for a total of 50 cells at least. (A) Quantitative analysis of FNDs, PLA-FND colocalization with early endosomes labeled with EEA1. The results indicate no statistically significant difference between the different groups. (B) Quantitative analysis of FNDs, PLA-FND colocalization with late endosomes and lysosomes labeled with LAMP-1. The results indicated statistically significant differences between FNDs or PLA-FNDs.

In order to further follow up on intracellular trafficking routes of particles, endosomes and lysosomes of macrophages were marked by EEA1 antibody and LAMP-1 antibody (see Figures 5 and 6). These organelles were chosen because endosomes and lysosomes are the main organelles through which internalized nanoparticles pass. We determined the proportion of FNDs and PLA-FNDs that colocalized with early endosomes EEA1 marked at 0, 6, and 24 h (see Figure 7). We found no statistically significant difference between 0 and 24 h for both FNDs and PLA-FNDs. For the FND group, the colocalization proportion with early endosomes was 19.43%, 22.79%, and 24.17% at 0 h, 6 h, and 24 h of incubation. FNDs are aggregated more at 6 h as a result of intracellular uptake. These results suggest that only a small part of FNDs were internalized in early endosomes, and most FNDs were already in the cytoplasm or late endosomes or lysosomes. The colocalization proportion of FNDs internalized in late endosomes or LMAP-1 marked lysosomes increased significantly over time from 27.27% for 0 h incubation to 44.32% for 6 h incubation and 51.07% for 24 h incubation. This indicates that the early endosomes where most FNDs were internalized have become late endosomes or lysosomes. It is possible that some FNDs escaped from endosomes to the cytoplasm and were translocated into lysosomes as the uptake time increased. It is also possible that a small part of the FNDs escaped from endosomes into the cytoplasm but did not translocate into lysosomes. Similar cases have been reported in some studies.^40,41^

For the PLA-FND group, the colocalization proportion of PLA-FNDs internalized at early endosomes is similar to FNDs. But, unlike FNDs, the proportion of PLA-FNDs colocalized with late endosomes or lysosomes, and LMAP-1 marked decreases significantly with incubation time. For 0 h incubation, internalization of PLA-FNDs was 26.52% in early endosomes, and internalization of PLA-FNDs was 56.75% in late endosomes and lysosomes. With the increase of incubation time, the proportion of PLA-FNDs internalized at early endosomes was still low at 6 h and 24 h incubation, reaching 22.36 and 19.63%, respectively. However, internalization of PLA-FNDs with late endosomes and lysosomes decreased to 42.35 and 34.56%. This result suggested that the early endosomes where PLA-FNDs were internalized had become late endosomes or lysosomes in the early uptake. It is likely that endosome or lysosome membranes were disrupted as uptake time increased since the endosome and lysosome environment is acidic with pH 5.0. PLA could be degraded in this environment, and then fragments will interact directly with the endosomal and lysosomal membrane and disrupt the membrane.^42^

### Recording FNDs and PLA-FND Trajectories in Living Cells

**Figure 8:**
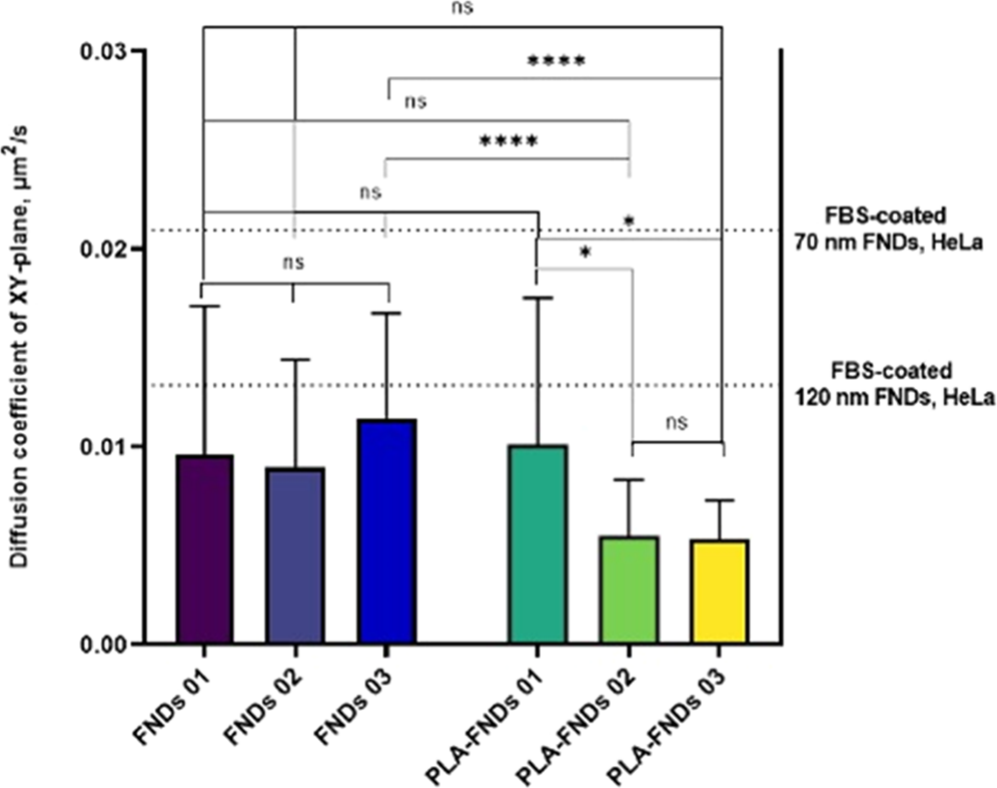
Mean diffusion coefficient during 8 h measurements in the *xy*-plane. Three FNDs and three PLA-FNDs were tested. Black dotted lines indicate the median diffusion coefficient of FBS-coated 70 and 120 nm FNDs in HeLa cells from previous work^43^ (different bare particles are numbered as FND 01, FND 02, and FND 03, while PLA-FND 01, PLA-FND 02, and PLA-FND 03 indicate FNDs that are in PLA particles).

In order to further explore the movement of particles inside cells, trajectories were recorded. Figure 8 shows the mean diffusion coefficient of 8 h trajectories on the *xy*-plane. We found diffusion coefficients much lower than those of FBS-coated 70 nm FNDs and 120 nm FNDs in HeLa cells. The behavior of PLA-FNDs 01 did not differ from those of the three FNDs tested, and PLA-FNDs 02 and 03 did not differ from FNDs 01 and 02 but differed significantly from FND 03, possibly because of differences between particles or the extent of aggregation of internalized particles. In addition, the movement trajectories of PLA-FNDs in the *xy*-plane did not change significantly over time (Figure S3 in Supporting Information). However, the volume that particles explored decreased more rapidly in FND-PLA compared with FNDs (see Figure S4 in the Supporting Information). Additionally, system drift could have a certain localization error, but there is not a massive impact on trajectories based on previous work.^43^ To summarize, the trajectory of a particle could be affected by many factors, and cells respond to different particle shapes and sizes, which influence trajectories.

## Conclusions

FNDs offer stable fluorescence and do not bleach or blink. As a result, they are attractive labels for long-term imaging and long-term tracking. Here, we have shown that these particles can be used to visualize, where polymer nanoparticles are transported within cells or tissues. More specifically, we were able to follow the uptake route in cells. We also quantified the number and volume of particles in the cell and evaluated the colocalization of the particles and organelles. In addition, PLA-FNDs have diffusion coefficients lower than FNDs. This indicates that either FNDs or aggregates or PLA-FNDs are transported differently. In tissues, we were able to observe PLA-FNDs using two-photon microscopy. While this work was limited to studying PLA fluorescent nanodiamonds, they can be applied to other polymer particles in the same fashion.

## Supporting Information Available

Protocols for nanodiamond tracking; characterization of PLA-FNDs, including SEM, DLS, confocal images; tracking results; XTT assay results to confirm the biocompatibility of particles; and details on quantitative image analysis (PDF)

## References

[ref1] Deirram N.; Zhang C.; Kermaniyan S. S.; Johnston A. P.; Such G. K. pH-responsive polymer nanoparticles for drug delivery. Macromol. Rapid Commun. 2019, 40 (10), 1800917 10.1002/marc.201800917. 30835923

[ref2] Feng X.; Lv F.; Liu L.; Tang H.; Xing C.; Yang Q.; Wang S. Conjugated polymer nanoparticles for drug delivery and imaging. ACS Appl. Mater. Interfaces 2010, 2 (8), 2429–2435. 10.1021/am100435k. 20695494

[ref3] Forier K.; Raemdonck K.; De Smedt S. C.; Demeester J.; Coenye T.; Braeckmans K. Lipid and polymer nanoparticles for drug delivery to bacterial biofilms. J. Controlled Release 2014, 190, 607–623. 10.1016/j.jconrel.2014.03.055. 24794896

[ref4] Sharifi S.; Behzadi S.; Laurent S.; Forrest M. L.; Stroeve P.; Mahmoudi M. Toxicity of nanomaterials. Chem. Soc. Rev. 2012, 41 (6), 2323–2343. 10.1039/C1CS15188F. 22170510 PMC4703119

[ref5] Paul M. B.; Stock V.; Cara-Carmona J.; Lisicki E.; Shopova S.; Fessard V.; Braeuning A.; Sieg H.; Böhmert L. Micro-and nanoplastics–current state of knowledge with the focus on oral uptake and toxicity. Nanoscale Adv. 2020, 2 (10), 4350–4367. 10.1039/D0NA00539H. 36132901 PMC9417819

[ref6] Mariano S.; Tacconi S.; Fidaleo M.; Rossi M.; Dini L. Micro and nanoplastics identification: classic methods and innovative detection techniques. Front. Toxicol. 2021, 3, 636640 10.3389/ftox.2021.636640. 35295124 PMC8915801

[ref7] Li G.; Yang Z.; Pei Z.; Li Y.; Yang R.; Liang Y.; Zhang Q.; Jiang G. Single-particle analysis of micro/nanoplastics by SEM-Raman technique. Talanta 2022, 249, 123701 10.1016/j.talanta.2022.123701. 35751923

[ref8] Shim W. J.; Song Y. K.; Hong S. H.; Jang M. Identification and quantification of microplastics using Nile Red staining. Mar. Pollut. Bull. 2016, 113 (1–2), 469–476. 10.1016/j.marpolbul.2016.10.049. 28340965

[ref9] Lv L.; He L.; Jiang S.; Chen J.; Zhou C.; Qu J.; Lu Y.; Hong P.; Sun S.; Li C. In situ surface-enhanced Raman spectroscopy for detecting microplastics and nanoplastics in aquatic environments. Sci. Total Environ. 2020, 728, 138449 10.1016/j.scitotenv.2020.138449. 32353796

[ref10] Vaijayanthimala V.; Cheng P. Y.; Yeh S. H.; Liu K. K.; Hsiao C. H.; Chao J. I.; Chang H. C. The long-term stability and biocompatibility of fluorescent nanodiamond as an in vivo contrast agent. Biomaterials 2012, 33 (31), 7794–7802. 10.1016/j.biomaterials.2012.06.084. 22863379

[ref11] Vaijayanthimala V.; Tzeng Y. K.; Chang H. C.; Li C. L. The biocompatibility of fluorescent nanodiamonds and their mechanism of cellular uptake. Nanotechnology 2009, 20 (42), 425103 10.1088/0957-4484/20/42/425103. 19779240

[ref12] Hsu T. C.; Liu K. K.; Chang H. C.; Hwang E.; Chao J. I. Labeling of neuronal differentiation and neuron cells with biocompatible fluorescent nanodiamonds. Sci. Rep. 2014, 4 (1), 5004 10.1038/srep05004. 24830447 PMC4023134

[ref13] van der Laan K.; Hasani M.; Zheng T.; Schirhagl R. Nanodiamonds for in vivo applications. Small 2018, 14 (19), 1703838 10.1002/smll.201703838. 29424097

[ref14] Haziza S.; Mohan N.; Loe-Mie Y.; Lepagnol-Bestel A. M.; Massou S.; Adam M. P.; Le X. L.; Viard J.; Plancon C.; Daudin R.; Koebel P.; et al. Fluorescent nanodiamond tracking reveals intraneuronal transport abnormalities induced by brain-disease-related genetic risk factors. Nat. Nanotechnol. 2017, 12 (4), 322–328. 10.1038/nnano.2016.260. 27893730

[ref15] Wu T. J.; Tzeng Y. K.; Chang W. W.; Cheng C. A.; Kuo Y.; Chien C. H.; Chang H. C.; Yu J. Tracking the engraftment and regenerative capabilities of transplanted lung stem cells using fluorescent nanodiamonds. Nat. Nanotechnol. 2013, 8 (9), 682–689. 10.1038/nnano.2013.147. 23912062 PMC7097076

[ref16] Sigaeva A.; Hochstetter A.; Bouyim S.; Chipaux M.; Stejfova M.; Cigler P.; Schirhagl R. Single-Particle Tracking and Trajectory Analysis of Fluorescent Nanodiamonds in Cell-Free Environment and Live Cells. Small 2022, 18 (39), 2201395 10.1002/smll.202201395. 36038355

[ref17] Hui Y. Y.; Hsiao W. W. W.; Haziza S.; Simonneau M.; Treussart F.; Chang H. C. Single particle tracking of fluorescent nanodiamonds in cells and organisms. Curr. Opin. Solid State Mater. Sci. 2017, 21 (1), 35–42. 10.1016/j.cossms.2016.04.002.

[ref18] Ghanimi Fard M.; Khabir Z.; Reineck P.; Cordina N. M.; Abe H.; Ohshima T.; Dalal S.; Gibson B. C.; Packer N. H.; Parker L. M. Targeting cell surface glycans with lectin-coated fluorescent nanodiamonds. Nanoscale Adv. 2022, 4 (6), 1551–1564. 10.1039/D2NA00036A. 36134370 PMC9418452

[ref19] Sharmin R.; Nusantara A. C.; Nie L.; Wu K.; Elias Llumbet A.; Woudstra W.; Mzyk A.; Schirhagl R. Intracellular Quantum Sensing of Free-Radical Generation Induced by Acetaminophen (APAP) in the Cytosol, in Mitochondria and the Nucleus of Macrophages. ACS Sens. 2022, 7 (11), 3326–3334. 10.1021/acssensors.2c01272. 36354956 PMC9706807

[ref20] Terada D.; Genjo T.; Segawa T. F.; Igarashi R.; Shirakawa M. Nanodiamonds for bioapplications–specific targeting strategies. Biochim. Biophys. Acta, Gen. Subj. 2020, 1864 (2), 129354 10.1016/j.bbagen.2019.04.019. 31071412

[ref21] Hemelaar S. R.; De Boer P.; Chipaux M.; Zuidema W.; Hamoh T.; Martinez F. P.; Nagl A.; Hoogenboom J. P.; Giepmans B. N. G.; Schirhagl R. Nanodiamonds as multi-purpose labels for microscopy. Sci. Rep. 2017, 7 (1), 720 10.1038/s41598-017-00797-2. 28389652 PMC5429637

[ref22] McGuinness L. P.; Yan Y.; Stacey A.; Simpson D. A.; Hall L. T.; Maclaurin D.; Prawer S.; Mulvaney P.; Wrachtrup J.; Caruso F.; Scholten R. E.; Hollenberg L. C. L. Quantum measurement and orientation tracking of fluorescent nanodiamonds inside living cells. Nat. Nanotechnol. 2011, 6 (6), 358–363. 10.1038/nnano.2011.64. 21552253

[ref23] Neumann P.; Jakobi I.; Dolde F.; Burk C.; Reuter R.; Waldherr G.; Honert J.; Wolf T.; Brunner A.; Shim J. H.; Suter D.; et al. High-precision nanoscale temperature sensing using single defects in diamond. Nano Lett. 2013, 13 (6), 2738–2742. 10.1021/nl401216y. 23721106

[ref24] Kucsko G.; Maurer P. C.; Yao N. Y.; Kubo M.; Noh H. J.; Lo P. K.; Park H.; Lukin M. D. Nanometre-scale thermometry in a living cell. Nature 2013, 500 (7460), 54–58. 10.1038/nature12373. 23903748 PMC4221854

[ref25] Tian Y.; Nusantara A. C.; Hamoh T.; Mzyk A.; Tian X.; Perona Martinez F.; Li R.; Permentier H. P.; Schirhagl R. Functionalized fluorescent nanodiamonds for simultaneous drug delivery and quantum sensing in HeLa cells. ACS Appl. Mater. Interfaces 2022, 14 (34), 39265–39273. 10.1021/acsami.2c11688. 35984747 PMC9437893

[ref26] Norouzi N.; Nusantara A. C.; Ong Y.; Hamoh T.; Nie L.; Morita A.; Zhang Y.; Mzyk A.; Schirhagl R. Relaxometry for detecting free radical generation during Bacteria’s response to antibiotics. Carbon 2022, 199, 444–452. 10.1016/j.carbon.2022.08.025.

[ref27] Wu K.; Nie L.; Nusantara A. C.; Woudstra W.; Vedelaar T.; Sigaeva A.; Schirhagl R. Diamond Relaxometry as a Tool to Investigate the Free Radical Dialogue between Macrophages and Bacteria. ACS Nano 2023, 17 (2), 1100–1111. 10.1021/acsnano.2c08190. 36630151 PMC9878971

[ref28] Reyes-San-Martin C.; Hamoh T.; Zhang Y.; Berendse L.; Klijn C.; Li R.; Llumbet A. E.; Sigaeva A.; Kawałko J.; Mzyk A.; Schirhagl R. Nanoscale mri for selective labeling and localized free radical measurements in the acrosomes of single sperm cells. ACS Nano 2022, 16 (7), 10701–10710. 10.1021/acsnano.2c02511. 35771989 PMC9331174

[ref29] Shenderova O. A.; Shames A. I.; Nunn N. A.; Torelli M. D.; Vlasov I.; Zaitsev A. Synthesis, properties, and applications of fluorescent diamond particles. J. Vac. Sci. Technol., B: Nanotechnol. Microelectron.: Mater., Process., Meas., Phenom. 2019, 37 (3), 030802 10.1116/1.5089898. PMC6461556 31032146

[ref30] Ong S. Y.; Van Harmelen R. J. J.; Norouzi N.; Offens F.; Venema I. M.; Najafi M. H.; Schirhagl R. Interaction of nanodiamonds with bacteria. Nanoscale 2018, 10 (36), 17117–17124. 10.1039/C8NR05183F. 30182122

[ref31] Jaiswal J.; Gupta S. K.; Kreuter J. Preparation of biodegradable cyclosporine nanoparticles by high-pressure emulsification-solvent evaporation process. J. Controlled Release 2004, 96 (1), 169–178. 10.1016/j.jconrel.2004.01.017. 15063039

[ref32] de Graaf I. A. M.; Olinga P.; De Jager M. H.; Merema M. T.; De Kanter R.; Van De Kerkhof E. G.; Groothuis G. M. Preparation and incubation of precision-cut liver and intestinal slices for application in drug metabolism and toxicity studies. Nat. Protoc. 2010, 5 (9), 1540–1551. 10.1038/nprot.2010.111. 20725069

[ref33] Foroozandeh P.; Aziz A. A. Insight into cellular uptake and intracellular trafficking of nanoparticles. Nanoscale Res. Lett. 2018, 13, 339 10.1186/s11671-018-2728-6. 30361809 PMC6202307

[ref34] van der Laan K. J.; Naulleau J.; Damle V. G.; Sigaeva A.; Jamot N.; Perona-Martinez F. P.; Chipaux M.; Schirhagl R. Toward using fluorescent nanodiamonds to study chronological aging in Saccharomyces cerevisiae. Anal. Chem. 2018, 90 (22), 13506–13513. 10.1021/acs.analchem.8b03431. 30345733

[ref35] Vaijayanthimala V.; Tzeng Y. K.; Chang H. C.; Li C. L. The biocompatibility of fluorescent nanodiamonds and their mechanism of cellular uptake. Nanotechnology 2009, 20 (42), 425103 10.1088/0957-4484/20/42/425103. 19779240

[ref36] Hemelaar S. R.; Saspaanithy B.; L’hommelet S. R.; Perona Martinez F. P.; Van der Laan K. J.; Schirhagl R. The response of HeLa cells to fluorescent nanodiamond uptake. Sensors 2018, 18 (2), 355 10.3390/s18020355. 29373504 PMC5855215

[ref37] Zipfel W. R.; Williams R. M.; Christie R.; Nikitin A. Y.; Hyman B. T.; Webb W. W. Live tissue intrinsic emission microscopy using multiphoton-excited native fluorescence and second harmonic generation. Proc. Natl. Acad. Sci. U.S.A. 2003, 100 (12), 7075–7080. 10.1073/pnas.0832308100. 12756303 PMC165832

[ref38] Jun Y. W.; Kim H. R.; Reo Y. J.; Dai M.; Ahn K. H. Addressing the autofluorescence issue in deep tissue imaging by two-photon microscopy: the significance of far-red emitting dyes. Chem. Sci. 2017, 8 (11), 7696–7704. 10.1039/C7SC03362A. 29568432 PMC5851340

[ref39] Li R.; Vedelaar T.; Mzyk A.; Morita A.; Padamati S. K.; Schirhagl R. Following Polymer Degradation with Nanodiamond Magnetometry. ACS Sens. 2022, 7 (1), 123–130. 10.1021/acssensors.1c01782. 34982542 PMC8809337

[ref40] Prabhakar N.; Khan M. H.; Peurla M.; Chang H. C.; Hänninen P. E.; Rosenholm J. M. Intracellular trafficking of fluorescent nanodiamonds and regulation of their cellular toxicity. ACS Omega 2017, 2 (6), 2689–2693. 10.1021/acsomega.7b00339. 30023673 PMC6044821

[ref41] Nie L.; Zhang Y.; Li L.; van Rijn P.; Schirhagl R. pH sensitive dextran coated fluorescent nanodiamonds as a biomarker for hela cells endocytic pathway and increased cellular uptake. Nanomaterials 2021, 11 (7), 1837 10.3390/nano11071837. 34361223 PMC8308332

[ref42] Selby L. I.; Cortez-Jugo C. M.; Such G. K.; Johnston A. P. Nanoescapology: progress toward understanding the endosomal escape of polymeric nanoparticles. Wiley Interdiscip. Rev.: Nanomed. Nanobiotechnol. 2017, 9 (5), e1452 10.1002/wnan.1452. 28160452

[ref43] Sigaeva A.; Hochstetter A.; Bouyim S.; Chipaux M.; Stejfova M.; Cigler P.; Schirhagl R. Single-Particle Tracking and Trajectory Analysis of Fluorescent Nanodiamonds in Cell-Free Environment and Live Cells. Small 2022, 18 (39), 2201395 10.1002/smll.202201395. 36038355

