# Supplementary material for: Fluorescent Nanodiamonds for Tracking Single Polymer Particles in Cells and Tissues
Source: Anal Chem. 2023 Aug 23;95(35):13046–54. doi: 10.1021/acs.analchem.3c01452 (PMC10483464; doi:10.1021/acs.analchem.3c01452)
Supplement: Supplementary file 1 — ac3c01452_si_001.pdf [file ac3c01452_si_001.pdf]

Supporting Information

Fluorescent Nanodiamonds for tracking single polymer particles in cells and tissues

Runrun Li[1], Thea A. Vedelaar[1], Alina Sigaeva[1], Yue Zhang[1], Kaiqi Wu[1], Hui Wang[2], Xixi Wu[1,2], Peter Olinga[3], Małgorzata K. Wlodarzyk-Biegun[2,4], Romana Schirhagl*[1]

1 Groningen University, University Medical Center Groningen, Department of Biomedical Engineering, Antonius Deusinglaan 1, 9713AV Groningen, The Netherlands
2 Groningen University, Zernike Institute for Advanced Materials, Nijenborgh 4, 9747 AG, Groningen, The Netherlands
3 Groningen University, University Medical Center Groningen, Department of Pharmaceutical Technology and Biopharmacy, Antonius Deusinglaan 1, 9713AV Groningen, The Netherlands
4 The Silesian University of Technology, Biotechnology Centre, Krzywoustego 8, 44-100 Gliwice, Poland

Email: romana.schirhagl@gmail.com

Nanodiamond tracking

Bare FNDs and PLA-FNDs were added to the J774 cells seeded in 35mm Petri dishes with a glass bottom and incubated for 2h. Then we replaced medium containing nanoparticles with fresh medium without particles. The sample was moved immediately to the setup and we started the experiment. Nanodiamond trajectories were recorded using a home-made confocal microscope, this system is similar to what is typically used in the diamond magnetometry community [1]. This home-made confocal microscope is only used to track nanodiamond movement. The specifications of this system and the tracking method were described in previous work [1]. 600nm long-pass filter was used to separate the FND signal from the background fluorescence. First, the algorithm took a 20×20 µm window at a single optical plane to observe cells with FNDs. Then FNDs were selected which were inside of cells. To localize the cells, we made use of the slight background fluorescence of the cell and the focused laser beam seen in the bright field from the same field of view. Next, we verified that a bright spot was indeed an FND that could be tracked. We chose particles with an average count per second rate above 1,000,000 (particles below that count rate are especially small) and below 4,000,000 (indicating a large aggregate). We further verified that the fluorescence intensity was stable. To locate the FND one 2 × 2 µm image was taken where the location of the FNDs was determined to be at the point with highest intensity in the x and y direction. Afterwards, we scanned 2 µm in z to find the point with the highest intensity. Then the trajectories of either bare FNDs or PLA-FNDs were recorded continuously for 8 hours. During this time, we repeated this algorithm 31579 times with 0.6 ms dwell time in xy and 1 ms in in the z axis. From these measurements we calculated diffusion coefficients (D, $\mu m^2/s$) in the xy-plane as well as the explored volume of PLA-FNDs and FNDs over time. Here we chose to analyse only the diffusion in xy due to the better resolution in xy compared to z. The trajectory analysis and the calculation of diffusion coefficients are described in our previous work [2].

Characterization of nanoparticles

To determine the size and morphology of PLA-FND we conducted SEM measurements. As shown in Fig. 2 A and B both PLA and PLA-FND have a rounded shape. For PLA this has been demonstrated before and thus was expected [3,4]. While FNDs themselves have not been embedded in PLA, many other types of particles or small molecules have been incorporated into PLA with similar incorporation results. It is also visible that PLA-FNDs are slightly larger than PLA. Furthermore, we observed barely any sharp-

edged diamond particles which indicates that nanodiamonds were well incorporated into PLA. Confocal images of PLA and PLA-FNDs particles (Fig. S1 in Supporting information) show that PLA have no visible red fluorescence. The red fluorescence of the PLA-FNDs coincided with the particles under the bright field, which also illustrates that FNDs were embedded in PLA.

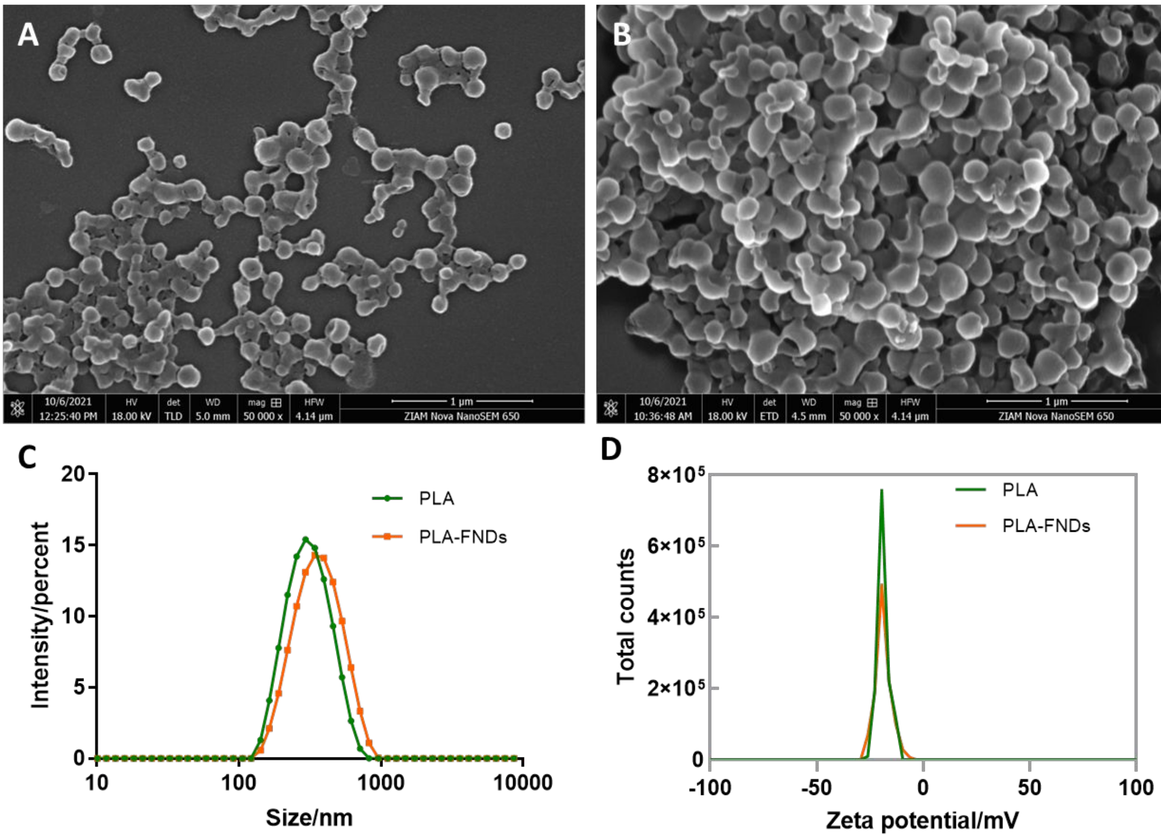

Fig.S1. Physical characterization of nanoparticles. Images of (A) PLA and (B) PLA-FNDs images obtained by SEM. (C) size distribution of PLA and PLA-FNDs. (D) Zeta potential distribution of PLA and PLA-FNDs.

This finding was also confirmed by dynamic light scattering measurements which reveals that PLA-FNDs have a hydrodynamic diameter of 348±94 nm and PLA of 325±114 nm. Further, we have determined the zeta potential of PLA and PLA-FND. It is also visible that both types of particles have a relatively narrow size distribution with PLA being slightly smaller than PLA-FND (see Table 1). This is an important parameter which reveals the particles colloidal stability. With 19±3 mv for PLA and -19±4 mv for PLA-FND the zeta potentials are almost identical. This means that both particle suspensions should be similarly colloidally stable in water. While nanodiamonds typically have a lower zeta potential of around -20 mv we saw only an insignificant decrease in zeta potential after adding FNDs to PLA. This is the case because we added a very small amount in order to retain the polymer properties. Further, the particles seem to incorporate the FNDs well.

Table S1. Parameters of PLA and PLA-FNDs nanoparticles suspensions in water

	**Particle size /nm**	**PDI**	**Zeta potential/mv**
PLA	325±114	0.107	-19±3
PLA-FND	348±94	0.211	-19±4

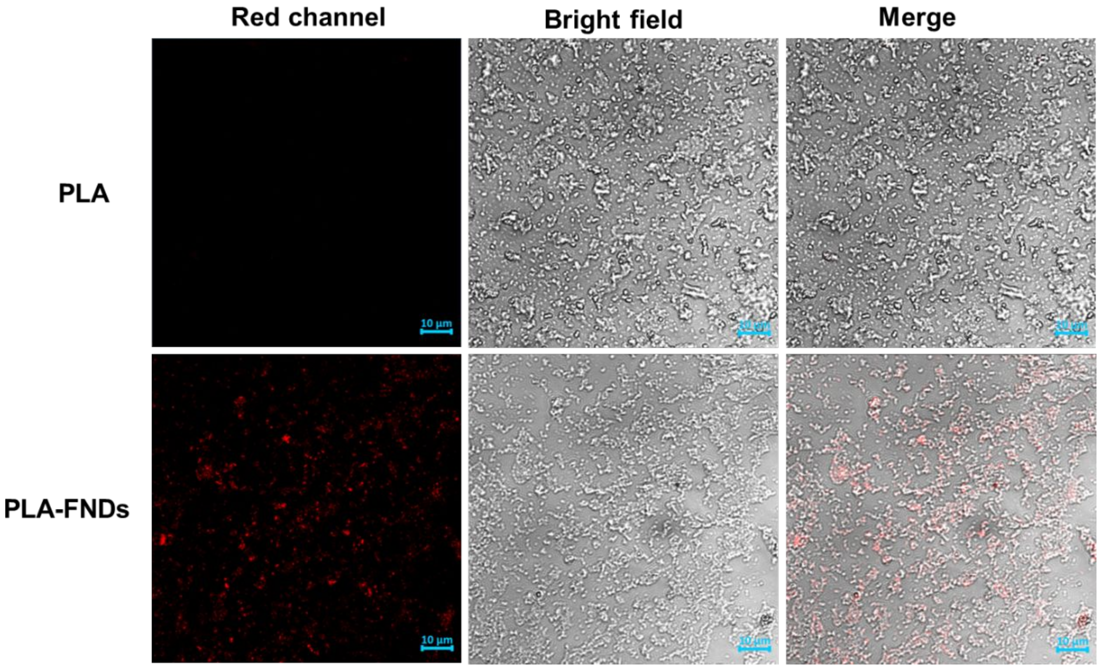

Fig.S2. The Comparison of PLA and PLA-FND confocal images. PLA particles have no fluorescence under the red channel, while PLA-FNDs have even red fluorescence dispersedly, which merged with the particles under the bright field.

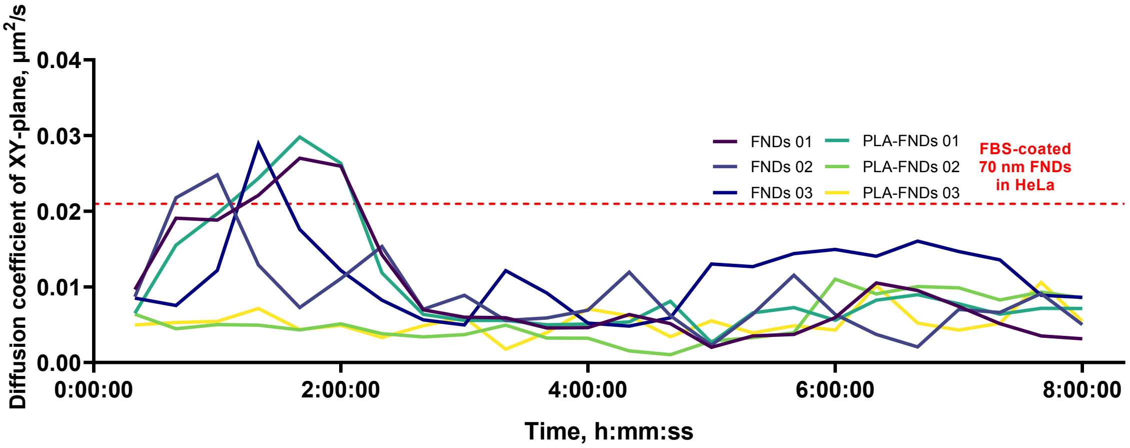

Fig.S3. Changes in diffusion coefficient of xy-plane of PLA-FNDs and 70 nm FNDs in J774 macrophages over time after 2h incubation. (green and yellow line: PLA-FNDs; blue and purple line: FNDs; red dot line: the median diffusion coefficient of FBS-coated 70 nm FNDs in HeLa)

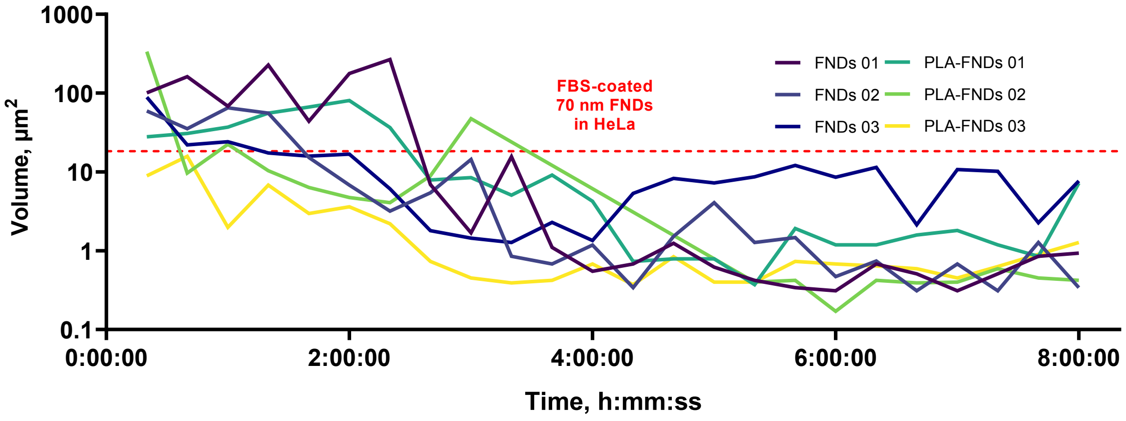

Fig.S4. Changes in the volume explored by PLA-FNDs and 70 nm FNDs in J774 macrophages over time. (green and yellow line: PLA-FNDs; blue and purple line: FNDs; red dot line: the median volume of FBS-coated 70 nm FNDs in HeLa)

Biocompatibility of PLA and PLA-FNDs

J774 cells were seeded in 96 well-plates with a cell density of 10000 cells/well, and then incubated for 48h in an incubator at 37℃ and 5% CO_2. An XTT cell proliferation kit was implemented using the manufacturer's procedure (ITW Reagents, Italy, A8088, 1000). In brief, experimental groups were 0 µg/mL, 50 µg/mL, 100 µg/mL, 200 µg/mL, 400 µg/mL of PLA and PLA-FNDs nanoparticles separately. As a background control we used cell medium without cells. Every group had six parallel wells. Cells were incubated with different concentrations of PLA and PLA-FNDs for 2h. Then we removed the medium containing particles, rinsed cells once with 1×PBS and added fresh medium. We then incubated for 0h, 6h, 24h. The XTT mixture was added into each well for 2h and we measured absorbance by a plate reader at 450 nm and 650 nm. The absorbance was determined by [Abs450nm (test)- Abs450nm (control)]- Abs660nm (test). Data were evaluated by GraphPad Prism 8.0. The statistics used the Kruskal-Wallis test of a one-way ANOVA.

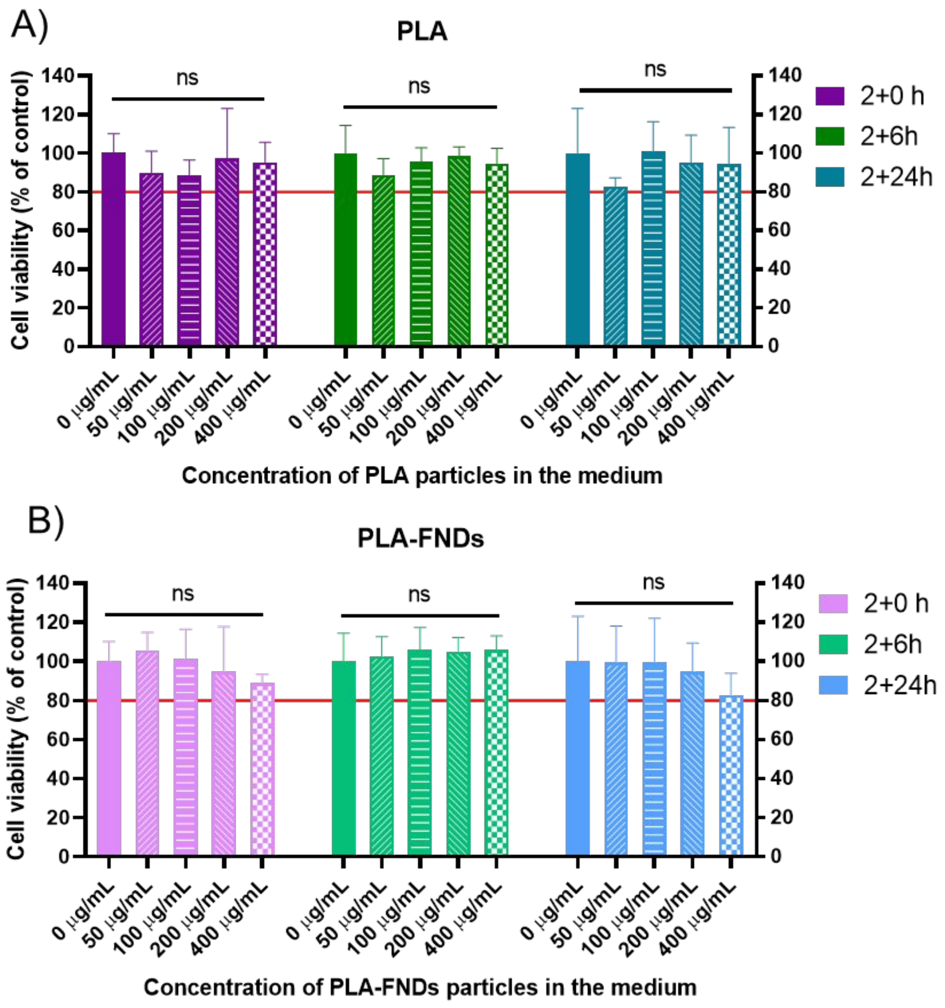

Figure S5, XTT assay performed after treatment with PLA and PLA-FND at different concentrations. (A) the cell viability after treatment with PLA particles. (B) the cell viability after treatment with PLA-FNDs. The red solid line represents the position of 80% cell viability (considered to be the normal range).

The XTT assay showed that the cell survival rate of cells incubated with both PLA and PLA-FNDs in the range of 50 μg/mL to 400 μg/mL was above 80% compared with the control cells. We didn’t see any significant differences in cell viability for any incubation conditions. Hence, we can conclude that 200 μg/mL of PLA and PLA-FNDs particles used to tack location in living cells had no cell toxicity.

Particle quantification

All images of cells were analyzed using FIJI software. To count the number of particles, we first selected a brightness threshold that allows us to identify individual particles while minimizing noise. Each pixel above this threshold was considered a particle. We then used the "3D Object Counter" plugin to obtain the total number of particles. For particle aggregates, if the brightness of an object exceeded a certain threshold, it was identified as an aggregate. The volume of these particle aggregates could be determined using the "Measure 3D" function of the "3D manager" plugin. To calculate colocalization, z-stack fluorescence images of different channels were transformed into a 3D Euclidean distance map of the marked protein. The distance between each particle and the marked protein was then calculated using the "3D Quantif" function of the "3D manager" plugin. If the minimum distance was 0, it indicated that

the particles were colocalized with the protein. The colocalization proportion was obtained by dividing the number of particles with a distance of 0 by the total number of internalized particles.

---

[1] Morita, A., Hamoh, T., Perona Martinez, F.P., Chipaux, M., Sigaeva, A., Mignon, C., van der Laan, K.J., Hochstetter, A. and Schirhagl, R., 2020. The fate of lipid-coated and uncoated fluorescent nanodiamonds during cell division in yeast. *Nanomaterials*, *10*(3), p.516.
[2] Sigaeva, A., Hochstetter, A., Bouyim, S., Chipaux, M., Stejfova, M., Cigler, P. and Schirhagl, R., 2022. Single-Particle Tracking and Trajectory Analysis of Fluorescent Nanodiamonds in Cell-Free Environment and Live Cells. *Small*, *18*(39), p.2201395.
[3] Kumari, A., Yadav, S.K., Pakade, Y.B., Kumar, V., Singh, B., Chaudhary, A. and Yadav, S.C., 2011. Nanoencapsulation and characterization of Albizia chinensis isolated antioxidant quercitrin on PLA nanoparticles. *Colloids and Surfaces B: Biointerfaces*, *82*(1), pp.224-232.
[4] Leo, E., Brina, B., Forni, F. and Vandelli, M.A., 2004. In vitro evaluation of PLA nanoparticles containing a lipophilic drug in water-soluble or insoluble form. *International journal of pharmaceutics*, *278*(1), pp.133-141.
